# Trastuzumab Emtansine for the Treatment of HER-2 Positive Carcinoma Ex-pleomorphic Adenoma Metastatic to the Brain: A Case Report

**DOI:** 10.3389/fonc.2018.00274

**Published:** 2018-07-24

**Authors:** Ihab Hassanieh, Lara Hilal, Karine A. Al Feghali, Ibrahim Khalifeh, Bassem Youssef

**Affiliations:** ^1^Department of Radiation Oncology, American University of Beirut, Beirut, Lebanon; ^2^Department of Pathology, American University of Beirut, Beirut, Lebanon

**Keywords:** carcinoma ex-pleomorphic adenoma, trastuzumab emtansine, brain metastases, radiotherapy, treatment

## Abstract

**Background:** Carcinoma Ex-pleomorphic adenoma is a malignant transformation of the common benign neoplasm of the salivary glands, “pleomorphic adenoma.” Only two cases were ever reported with brain metastases, with absence of good evidence guiding management of such cases.

**Case Presentation:** A 61-year-old woman presenting with facial paralysis was found to have carcinoma ex-pleomorphic adenoma of the parotid gland. Twenty months after local treatment, she developed brain metastases, treated with whole brain radiation therapy. The patient then had progressive intracranial disease after the end of radiation therapy in addition to the appearance of liver metastases. Pathology showed overexpression of HER2, so she was treated with Trastuzumab Emtansine (TDM1). Follow-up imaging revealed significant decrease in the number and size of the metastatic brain lesions in keeping with a good response to TDM1 treatment.

**Conclusion:** Prognosis of metastatic carcinoma ex-pleomorphic adenoma is very poor, and there is no clear management for such cases. We present a case of carcinoma ex-pleomorphic adenoma with brain and liver metastases with a very good response to TDM1 treatment.

## Background

Carcinoma Ex-pleomorphic adenoma (CXPA) is a malignant transformation of the common benign neoplasm of the salivary glands, “pleomorphic adenoma.” Even though pleomorphic adenoma is the most common benign primary salivary gland neoplasm, its malignant counterpart carcinoma ex-pleomorphic adenoma is uncommon, and has a prevalence rate of 5.6 cases per 100,000 malignant salivary gland neoplasms (1). Also, only one third of these tumors are thought to be metastatic (2). Systemic treatment options for metastatic CXPA are not yet clearly defined. Targeted therapy using drugs that target the human epidermal growth factor receptor 2 (HER2) such as Trastuzumab has been reported in few cases of CXPA with HER2 protein expression (3). However, only two cases were ever reported with brain metastases (4, 5). The first report of carcinoma ex-pleomorphic adenoma with metastasis to the brain dates back to 2006 (4). We are thus reporting the third case of carcinoma ex-pleomorphic adenoma metastastatic to the brain, with significant response to radiation and systemic therapy using Trastuzumab emtansine (TDM1).

## Case presentation

A 61-year-old woman presented in September 2014 for workup of a 4-month history of progressive right facial palsy, associated with progressively worsening right facial pain. She reported a history of pleomorphic adenoma, first resected in 1973 with 2 recurrences and excisions in 1993 and 2003.

On physical examination, there was a 2 × 2 cm hard, immobile, right sided preauricular mass, associated with multiple palpable level II lymph nodes. Cranial nerves (CN) were intact bilaterally except for complete right CN VII palsy.

Neck MRI revealed a multiloculated cystic lesion involving the superficial and deep lobes of the right parotid gland, and extending posteriorly to the retromandibular vein measuring 3.5 cm. There were six satellite nodules superficial to the right sternocleidomastoid muscle (SCM), highly suspicious for seeding of pleomorphic adenoma, the largest measuring 6 cm. Subsequent fine needle aspirate (FNA) showed pleomorphic adenoma. The patient underwent right parotidectomy, resection of the satellite nodules, and right level II lymph node dissection.

**Figure 1:**
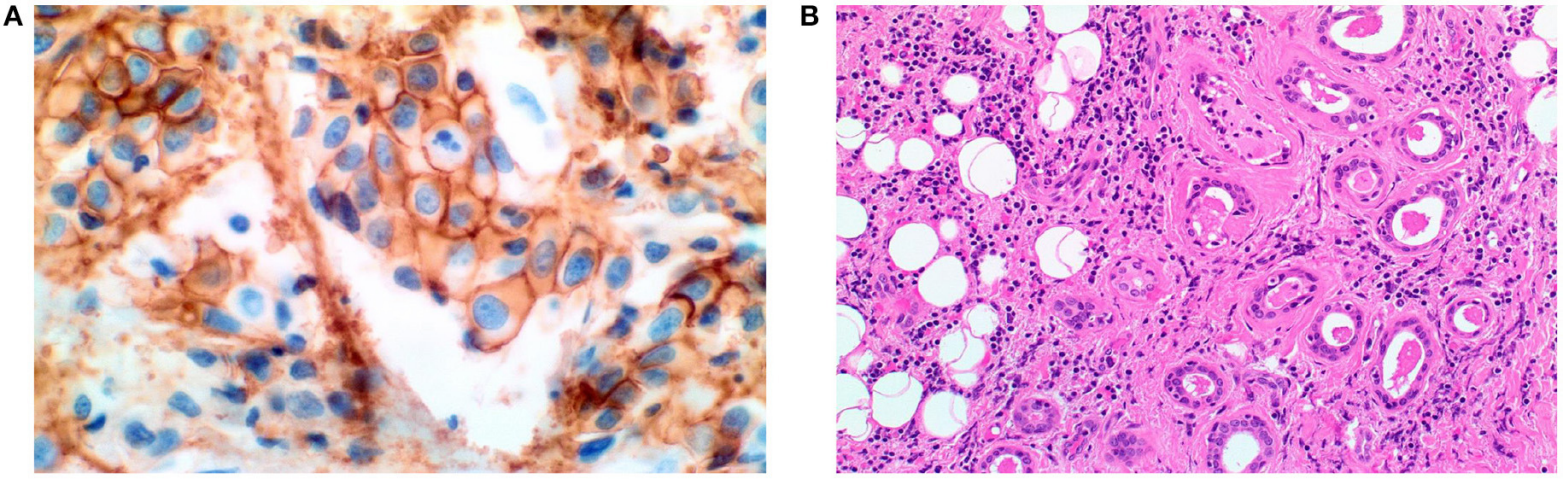
(200X magnification). **(A)** HER-2 immunostain showing overexpression of HER-2 with intense circumferential staining of the tumor cells. **(B)** Hematoxylin and Eosin stained section showing malignant glands infiltrating adipose tissue.

Pathology revealed carcinoma ex-pleomorphic adenoma, with positive cytokeratin (CK) 7 and negative CK5/6, CK20, P63, and thyroid transciption factor 1 (TTF-1). Two out of the six dissected lymph nodes were positive for carcinoma with no extra-capsular extension. However, there was perineural invasion as well as involvement of the SCM (Figure 1).

The patient then underwent right radical parotidectomy with modified radical neck dissection, including levels IA, IB, IIA, IIB, III, IV, and VA, as well as right lateral temporal bone resection. Pathology also revealed carcinoma ex-pleomorphic adenoma involving fibroelastic tissue and skeletal muscles with positive margins. Three out of 13 lymph nodes were involved by carcinoma. There was also vascular invasion with tumor involving both the jugular vein and the internal carotid artery.

Postoperative course was smooth except for a minor post-operative infection treated with intravenous antibiotics. The patient then received adjuvant radiation therapy, to a total dose of 66 Gray (Gy) in 33 fractions using Intensity modulated radiation therapy (IMRT) with simultaneous integrated boost (SIB). Radiotherapy course was completed on December 2014.

The patient was then followed up with routine imaging. In August 2016, Brain MRI showed evidence of more than thirty small lesions suggestive of brain metastases. Positron emission tomography (PET) scan showed no evidence of systemic disease.

The patient received 30 Gy in 12 fractions to the whole brain, limiting the dose to the previously irradiated region to 20 Gy, using IMRT, completed in August 2016. Follow up Brain MRI 2 months after radiation therapy showed resolution of the majority of the brain metastatic lesions with only few remaining visible lesions. Follow up Brain MRI 10 weeks later showed progression of the metastatic brain disease with at least 10 visible lesions. PET scan showed evidence of a metastatic liver lesion.

**Figure 2:**
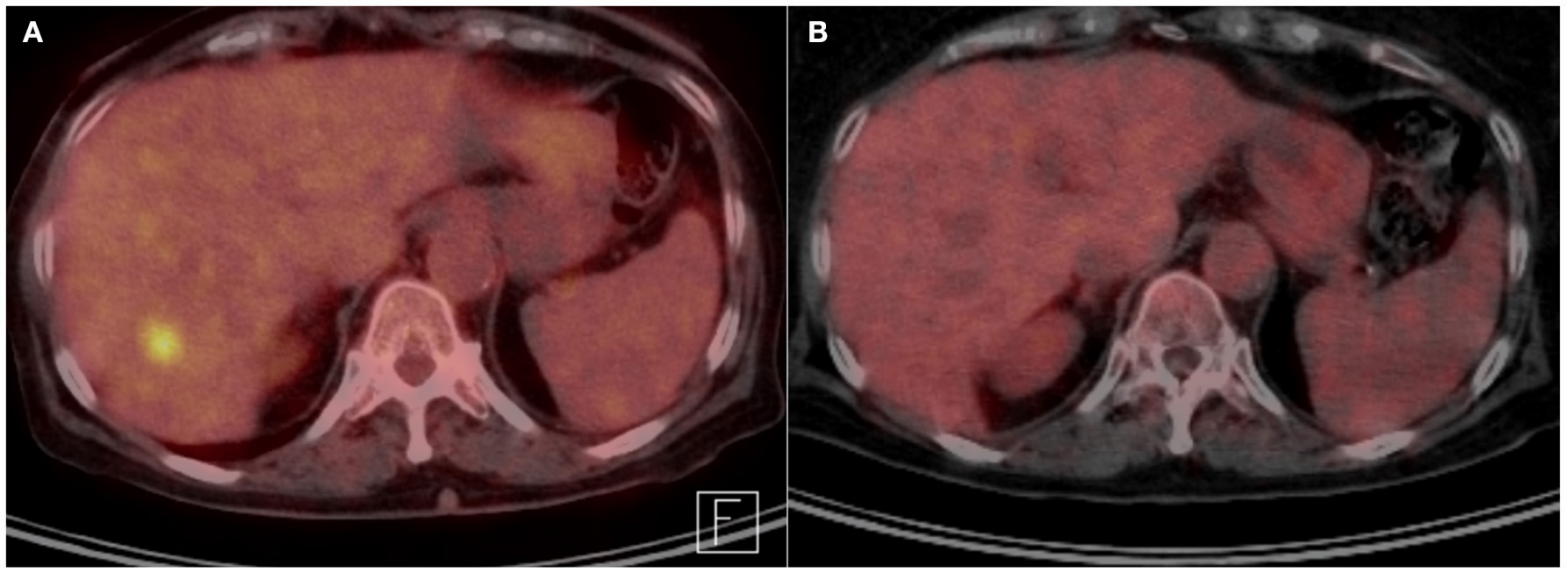
**(A)** Transverse abdominal PET scan before systemic therapy with TDM1, showing FDG avid liver lesion. **(B)** Transverse abdominal PET scan after treatment, showing disappearance of FDG avid liver lesion.

**Figure 3:**
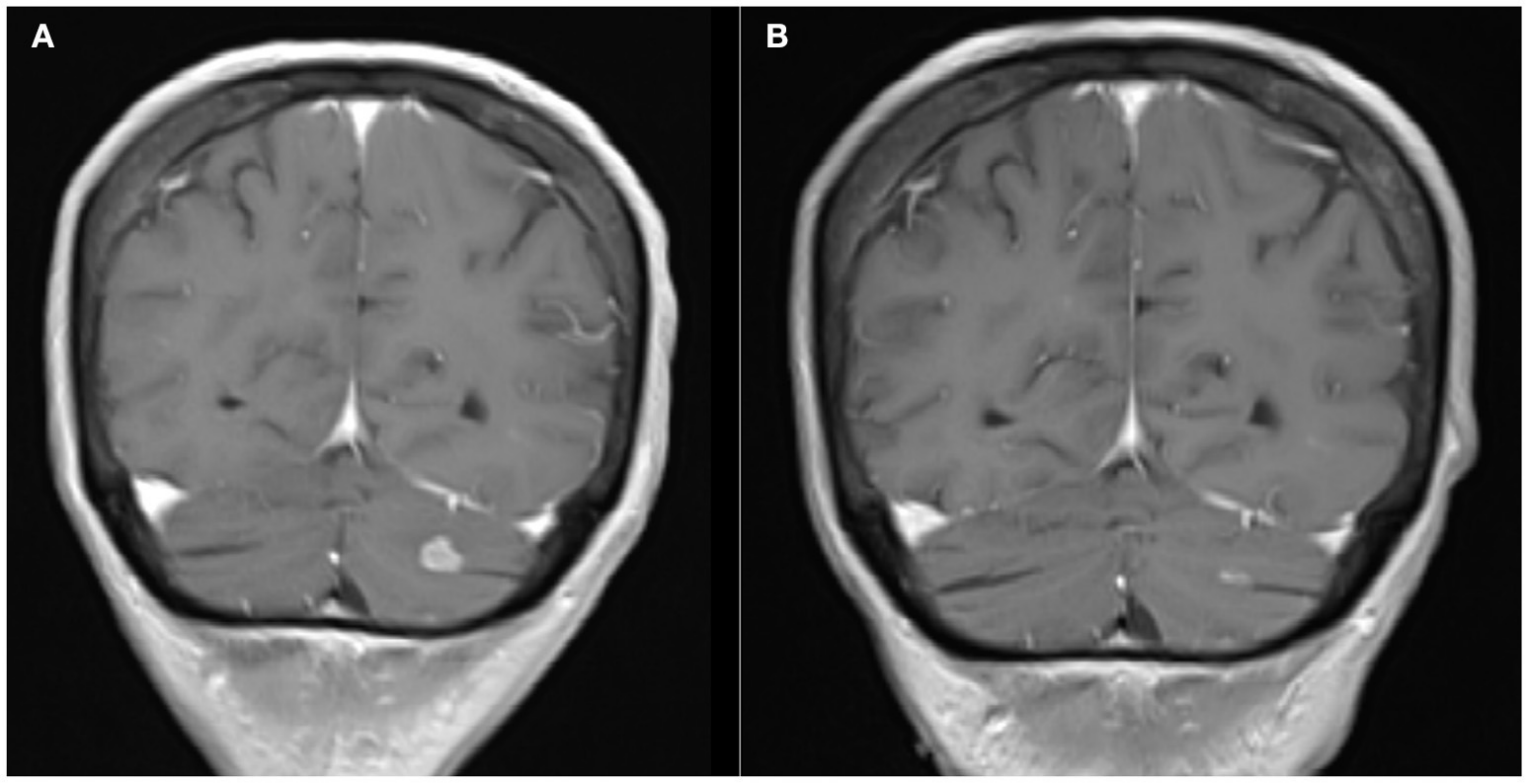
**(A)** Coronal Brain MRI, T1 with contrast showing a hyperintense lesion in the left cerebellar lobe before systemic therapy with TDM1. **(B)** Coronal Brain MRI, T1 with contrast showing decrease in the size of the lesion after treatment.

The patient was referred to medical oncology for consideration of systemic treatment. HER-2 staining was performed on the previous surgical specimen and showed overexpression of HER-2 (Figure 1). She received four cycles of Trastuzumab Emtansine (TDM1). Follow up PET scan four months later, showed resolution of the liver metastatic lesion (Figure 2). MRI brain revealed a significant decrease in the number and size of the metastatic enhancing brain lesions, in keeping with a good response to treatment (Figure 3).

## Discussion

Brain metastasis from Carcinoma ex-pleomorphic adenoma is rare. Those patients are usually managed with brain radiation therapy (RT). There is lack of data on the management of patients diagnosed with carcinoma ex-pleomorphic adenoma with progressive intracranial disease after RT. Multiple recent studies have shown expression of HER-2 to be common in carcinoma ex-pleomorphic adenoma occurring in 30-54% of cases (6, 7). Anti-HER2 therapy,trastuzumab has been used in the treatment of metastatic carcinoma ex-pleomorphic adenoma. In one report it was shown to cause remission in an advanced case of CXPA with bone metastasis (8). In another report, complete response was also achieved in a patient with CXPA metastatic to the lungs after treatment with Trastuzumab based chemotherapy (9).

In two previous case reports on metastatic CXPA to the brain, the patients passed away after 2 and 18 months, respectively (4, 5). In the first case, disease progressed aggressively and the patient passed away before systemic therapy was initiated (4). In the second case, there was a solitary large brain metastasis which was surgically resected followed by adjuvant RT and chemotherapy. Targeted therapy was not given since HER2 was not over expressed (5).

Our case illustrates an excellent response of Carcinoma ex-pleomorphic adenoma to Trastuzumab emtansine (TDM1) whereby our patient had resolution of her liver metastasis and significant intracranial disease response.This represents a possible treatment option for metastatic Carcinoma ex pleomorphic adenoma with HER2 overexpression. It can also be used in the setting of progressive brain metastases after RT. Although the blood-brain barrier is not permeable to large molecules like trastuzumab, recent reports have shown that this barrier might be disrupted in the setting of brain metastases, allowing the crossing of TDM1 (10). This could explain the good response achieved with the use of TDM1 in our patient.

## Conclusion

Prognosis of metastatic carcinoma ex-pleomorphic adenoma is very poor, and there is no clear management for such cases. We present a case of carcinoma ex-pleomorphic adenoma with brain and liver metastases with very good response to TDM1 treatment. In the absence of level I evidence guiding management in such cases, this is a good treatment option to consider.

## Ethics statement

This case report was carried out with the approval of the treated patient. The patient gave written informed consent in accordance with the Declaration of Helsinki.

## Author contributions

BY: supervised the writing of the paper and patient treatment. IH: writing manuscript and communication with patient. LH: manuscript review. KA: manuscript review, resident in charge of patient during treatment. IK: provided pathology report and images.

### Conflict of interest statement

The authors declare that the research was conducted in the absence of any commercial or financial relationships that could be construed as a potential conflict of interest.
